# Dimeric Ankyrin with Inverted Module Promotes Bifunctional Property in Capturing Capsid to Impede HIV-1 Replication

**DOI:** 10.3390/ijms24065266

**Published:** 2023-03-09

**Authors:** On-anong Juntit, Kanokporn Sornsuwan, Tanchanok Wisitponchai, Vannajan Sanghiran Lee, Supachai Sakkhachornphop, Umpa Yasamut, Chatchai Tayapiwatana

**Affiliations:** 1 Division of Clinical Immunology, Department of Medical Technology, Faculty of Associated Medical Sciences, Chiang Mai University, Chiang Mai 50200, Thailand; onanong_jun@cmu.ac.th (O.-a.J.); kanokporn_sornsuwan@cmu.ac.th (K.S.); miisuii@hotmail.com (T.W.); umpa.yas@cmu.ac.th (U.Y.); 2 Center of Biomolecular Therapy and Diagnostic, Faculty of Associated Medical Sciences, Chiang Mai University, Chiang Mai 50200, Thailand; 3 Department of Chemistry, Drug Design Development Research Group, Center of Theoretical and Computational Physics, Faculty of Science, University of Malaya, Kuala Lumpur 50603, Malaysia; vannajan@um.edu.my; 4 Research Institute for Health Sciences, Chiang Mai University, Chiang Mai 50200, Thailand; supachai.sak@cmu.ac.th; 5 Center of Innovative Immunodiagnostic Development, Faculty of Associated Medical Sciences, Chiang Mai University, Chiang Mai 50200, Thailand

**Keywords:** HIV-1 assembly, dimeric DARPin, inverted ankyrin, replication inhibitor, HIV-1 drug-resistant

## Abstract

Several anti-HIV scaffolds have been proposed as complementary treatments to highly active antiretroviral therapy. Ank^GAG^1D4, a designed ankyrin repeat protein, formerly demonstrated anti-HIV-1 replication by interfering with HIV-1 Gag polymerization. However, the improvement of the effectiveness was considered. Recently, the dimeric molecules of Ank^GAG^1D4 were accomplished in enhancing the binding activity against HIV-1 capsid (CAp24). In this study, the interaction of CAp24 against the dimer conformations was elucidated to elaborate the bifunctional property. The accessibility of the ankyrin binding domains was inspected by bio-layer interferometry. By inverting the second module of dimeric ankyrin (Ank^GAG^1D4_NC-CN_), the CAp24 interaction K_D_ was significantly reduced. This reflects the capability of Ank^GAG^1D4_NC-CN_ in simultaneously capturing CAp24. On the contrary, the binding activity of dimeric Ank^GAG^1D4_NC-NC_ was indistinguishable from the monomeric Ank^GAG^1D4. The bifunctional property of Ank^GAG^1D4_NC-CN_ was subsequently confirmed in the secondary reaction with additional p17p24. This data correlates with the MD simulation, which suggested the flexibility of the Ank^GAG^1D4_NC-CN_ structure. The CAp24 capturing capacity was influenced by the distance of the Ank^GAG^1D4 binding domains to introduce the avidity mode of Ank^GAG^1D4_NC-CN_. Consequently, Ank^GAG^1D4_NC-CN_ showed superior potency in interfering with HIV-1 NL4-3 WT and HIV-1 NL4-3 MIR_CAI201V_ replication than Ank^GAG^1D4_NC-NC_ and an affinity improved Ank^GAG^1D4-S45Y.

## 1. Introduction

Non-immunoglobulin scaffolds have been established as promising alternatives to overcome the limitation of monoclonal antibodies. Protein scaffolds offer architecture, lack of disulfide, high solubility, and differential molecular weight, allowing for unique biophysical and pharmacokinetic properties. Scaffold-based platforms are versatile synthetic binders demonstrating many therapeutic prospects, such as quinoline and its derivatives, demonstrating many therapeutic prospects. Quinoline hybrids, a versatile pharmacophore, are clinically used as an antitumor, anti-inflammatory, and anti-human immunodeficiency virus type 1 (HIV-1) reverse transcription. Certain proteins are currently attracting attention for the interference of HIV-1 replication. The potential of designed ankyrin-repeat proteins (DARPins) has been proposed as an alternative treatment to monoclonal antibodies [1,2,3]. Ank^GAG^1D4 is an intracellular anti-HIV-1 DARPin that specifically targets HIV-1 capsid CAp24 [4]. This Gag-specific artificial ankyrin inhibits HIV-1 virion production in the late-stage HIV-1 life cycle. Additionally, the ability of Ank^GAG^1D4 to disturb gag distribution in infected cells and interfere with RNA packaging of HIV-1 virion was demonstrated [5]. However, the HIV-1 protection by Ank^GAG^1D4 in long-term culture is insufficient. Although this phenomenon may not represent actual infection, improving the binding activity of Ank^GAG^1D4 against HIV-1 CAp24 should be considered.

Previously, the affinity of Ank^GAG^1D4 in targeting CAp24 was improved. The amino acid residue of Ank^GAG^1D4 was modified by substitution serine (S) for tyrosine (Y) at position 45 to increase the binding affinity. According to the bio-layer interferometry (BLI) experiment, Ank^GAG^1D4-S45Y mutant successfully improved the binding affinity compared to Ank^GAG^1D4 [6]. Likewise, Ank^GAG^1D4-S45Y markedly inhibits HIV-1 wild-type and the HIV-1 maturation inhibitor-resistant (MIR) strain in SupT1 cells [7]. However, viral escape was observed in late infection. Apart from mutagenesis, the construction of multimers is an alternative strategy. Bispecific-epidermal growth factor receptor (EGFR) DARPin with subnanomolar affinity dramatically reduced surface EGFR levels by inhibiting A431 cell proliferation and receptor recycling [8]. Recently, trimeric DARPins FSR16m and FSR22 efficiently block the SARS-CoV-2 virus entry by binding the RBD with high avidity compared to monomeric DARPin SR16m and SR22 [9]. Furthermore, tri-specific DARPin, Ensovibep, recognized the three units of the trimeric SARS-CoV-2 spike protein with picomolar range promising clinical candidate [10]. In reliance on this strategy, dimeric Ank^GAG^1D4 (Ank^GAG^1D4_NC-NC_ and Ank^GAG^1D4_NC-CN_) was synthesized and demonstrated improved avidity against CAp24. The binding activity of Ank^GAG^1D4_NC-CN_ was remarkably improved compared to Ank^GAG^1D4_NC-NC_ and parental Ank^GAG^1D4 [11]. A design strategy described in this study involved inverting the second module in Ank^GAG^1D4_NC-CN_ to promote the bifunctional property when targeting a large molecule. The biological function of dimeric Ank^GAG^1D4 in interfering with HIV-1 replication has yet to be elucidated.

The challenge in HIV-1 treatment is emerging of ART-resistant mutation. Several alternative anti-HIV-1 inhibitors were researched to counteract this issue. Due to a high-level sequence conservation, HIV-1 CAp24 is a promising target for an anti-HIV-1 strategy [12,13]. HIV-1 maturation inhibitor (MI) is a class of anti-HIV-1 compounds that block HIV-1 Gag processing at the CA-SP1 junction, resulting in an immature HIV-1 virion production [14,15]. However, the mutation in a target region of MI was reported [16,17,18]. A mutation at the HIV-1 C-terminus of the capsid (CA-CTD), isoleucine (I) position 201 to valine (V), results in PF46396 resistance and partial resistance against Bevirimat [16]. Since the mutation occurs in the CA-CTD unrelated to the target region of ankyrin, dimeric ankyrins are presumed to interfere with HIV-1 MIR_CAI201V_ virus production.

This study demonstrated an intracellular anti-HIV-1 activity of dimeric Ank^GAG^1D4 in HIV-1 infected SupT1 cells. Additionally, the antiviral activity of dimeric Ank^GAG^1D4 was investigated in HIV-1 CA-mutation strains. HIV-1 NL4-3 MIR_CAI201V_ was the representative model for the HIV-1 MIR virus. In contrast to monomeric Ank^GAG^1D4-S45Y, our results indicate an improvement of dimeric Ank^GAG^1D4 in effectively inhibiting HIV-1 replication.

## 2. Results

### 2.1. Inversion of Ank^GAG^1D4 Structure Does Not Alter Ankyrin Binding Surface

**Figure 1:**
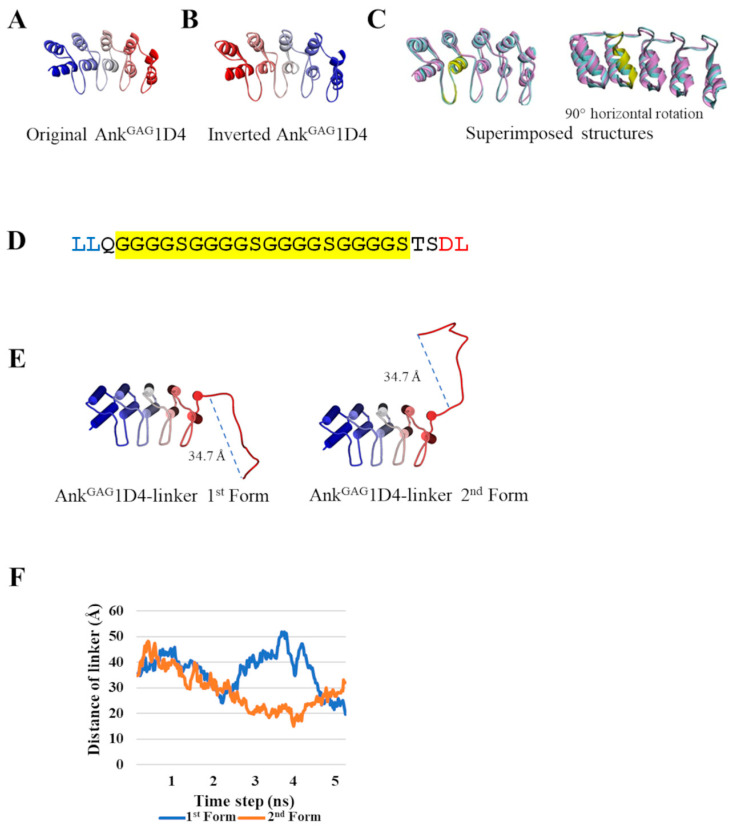
Initial structure of ankyrin before creating Ank^GAG^1D4 dimers: (**A**) The structure of the original Ank^GAG^1D4; (**B**) Inverted Ank^GAG^1D4, which is presented in ribbon style from blue at the N-terminus to red at the C-terminus; (**C**) The superimposed structure between the original Ank^GAG^1D4 (pink) and inverted Ank^GAG^1D4 (sky) with the yellow showing binding region, 44 DSIGSTPLHLAAYYG 58; (**D**) The amino sequence of the flexible linker consists of (G_4_S)_4_ linker and extra residues. Blue and red are used for superimposition with the first and second ankyrin modules, respectively; (**E**) Two versions of Ank^GAG^1D4-linker were obtained from superimposition between Ank^GAG^1D4 and a linker. They are initial structures for molecular dynamics simulation. The blue dash line shows how linker distance is measured; (**F**) Distance of the linker is part of the simulated dynamics of the Ank^GAG^1D4-linker during 5 ns.

The homology structure of the inverted Ank^GAG^1D4 was constructed with the same backbone torsional angles as the original Ank^GAG^1D4 (Figure 1A,B). However, these amino acids did not participate in the crucial helix compartments of the ankyrin architecture. Notably, the overall superimposed structure of inverted Ank^GAG^1D4 was not significantly different from its parent structure since the root-mean-square deviation (RMSD) of alpha carbons was at 0.2 Å (Figure 1C). These findings implied that the binding surface of the inverted Ank^GAG^1D4 was retained. In vitro, Ank^GAG^1D4_NC-CN_ harbored double-binding domains. The structure of inverted Ank^GAG^1D4 was linked with another ankyrin module to determine dimeric conformity.

### 2.2. Ank^GAG^1D4 Dimers Exhibit Diverse Molecular Structures

**Figure 2:**
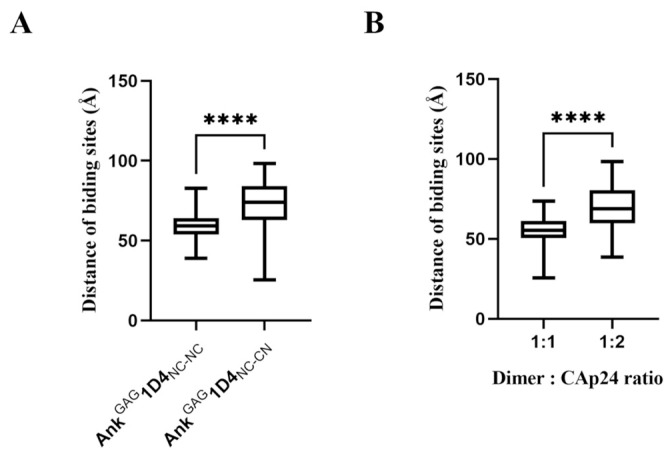
Distance of binding sites of Ank^GAG^1D4_NC-NC_ and Ank^GAG^1D4_NC-CN_: (**A**) The binding sites distance of Ank^GAG^1D4_NC-NC_ with 59.3 ± 7.5 Å (*n* = 397) and Ank^GAG^1D4_NC-CN_ with 71.9 ± 15.0 Å (*n* = 403); (**B**) The binding sites distance of Ank^GAG^1D4_NC-NC_ and Ank^GAG^1D4_NC-CN_ show 1:1 interaction at 54.8 ± 9.1 Å (*n* = 217) and 1:2 interaction at 69.7 ± 12.6 Å (*n* = 583). This data denote mean ± SD. **** *p* < 0.0001 using two-tailed unpaired *t*-test.

**Figure 3:**
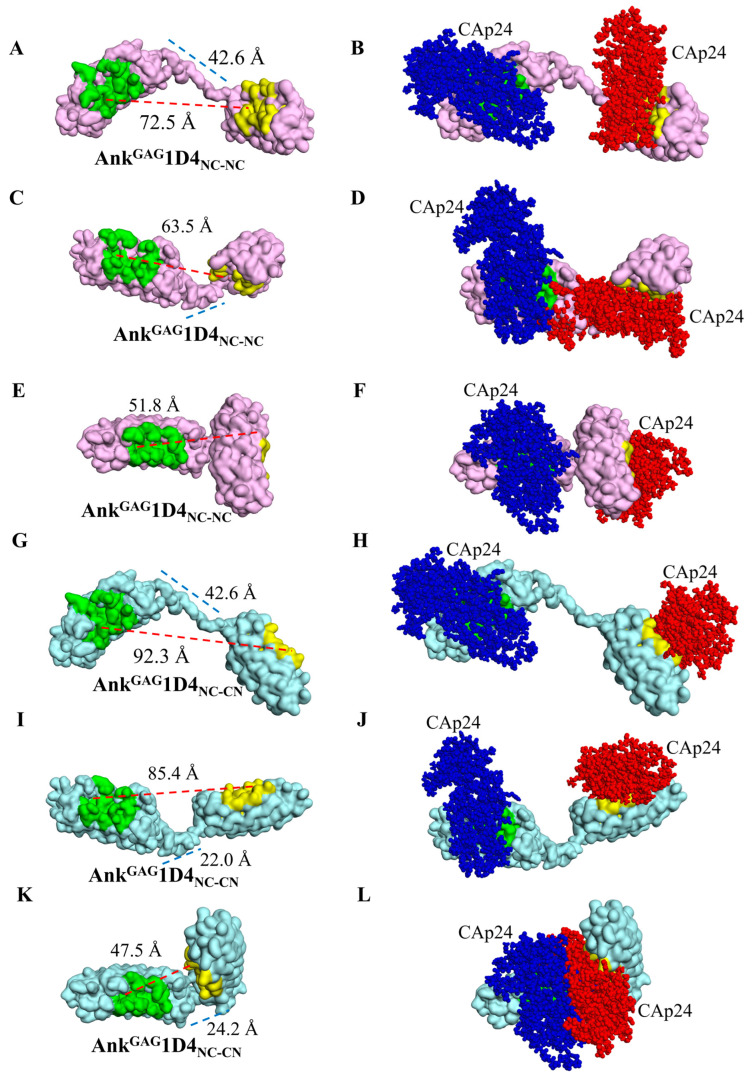
Characteristics of a conformational variant of Ank^GAG^1D4_NC-NC_ and Ank^GAG^1D4_NC-CN_. Examples of ankyrin dimers were selected with a range of linker distance (blue dashed line) and binding sites distance (red line). The surface style of Ank^GAG^1D4_NC-NC_ (pink) is shown in binding sites distances ranging from (**A**) long, (**C**) medium, and (**E**) short. Ank^GAG^1D4_NC-CN_ (sky) structures are presented in panels (**G**,**I**,**K**) with the same pattern. Panels (**B**,**D**,**F**) are the superimpositions between the docked structure Ank^GAG^1D4-CAp24 and the Ank^GAG^1D4_NC-NC_, compared with that of the Ank^GAG^1D4_NC-CN_ in panels (**H**,**J**,**L**). The CAp24 binding to the first module is colored blue, and that of the second module is red. Panels (**D**,**L**) present a 1:1 interaction, whereas panels (**B**,**F**,**H**,**J**) are a 1:2 interaction.

Ankyrin fusion proteins were constructed to validate their binding properties against CAp24. Initially, Ank^GAG^1D4, in conjunction with a flexible linker (Figure 1D) at its C-terminus (the Ank^GAG^1D4-linker), was simulated based on molecular dynamics at 5 ns to observe the dynamic behavior of the interdomain linker in ankyrin dimerization. The result for the Ank^GAG^1D4-linker (Figure 1E) revealed a diversifying conformation with the head-to-tail linker distance ranging between 15.0 and 51.9 Å (Figure 1F). The C-termini of 500 snapshots of molecular dynamics Ank^GAG^1D4-linker were extracted from 0–5 ns simulations and connected with Ank^GAG^1D4 to generate the second module of Ank^GAG^1D4_NC-NC_. Using a similar procedure, these Ank^GAG^1D4-linkers were assembled with an inverted Ank^GAG^1D4 to obtain Ank^GAG^1D4_NC-CN_. The majority of the Ank^GAG^1D4-linkers (403 of 500) displayed nonoverlapping coordinates with the second ankyrin module in both Ank^GAG^1D4_NC-NC_ and Ank^GAG^1D4_NC-CN_. The active binding sites of Ank^GAG^1D4_NC-CN_ (71.9 ± 15.0 Å) were more separate than Ank^GAG^1D4_NC-NC_ (59.3 ± 7.5 Å) (Figure 2A). The ratio of ankyrin dimers to CAp24s interaction depends on the distance between binding domains. The distance of 1:1 and 1:2 interactions were 54.8 ± 9.1 Å and 69.7 ± 12.6 Å, respectively (Figure 2B). Moreover, the conformational variability of the modeled dimers was readily apparent from the exemplifications (Figure 3). A variety of distances of Ank^GAG^1D4_NC-NC_ and Ank^GAG^1D4_NC-CN_ ranged from long (Figure 3A,C), medium (Figure 3C,I) to short (Figure 3E,K). Interestingly, with the same topology as the Ank^GAG^1D4-linker, the direction of the binding surface on the second ankyrin module of Ank^GAG^1D4_NC-NC_ and Ank^GAG^1D4_NC-CN_ differed. For example, the Ank^GAG^1D4_NC-NC_ allowed the two binding sites to face in the same direction (Figure 3A,C) in a “clam-shaped structure” conformation. However, the binding surfaces of Ank^GAG^1D4_NC-CN_ turned in the opposite direction (Figure 3G,I). The “clam-shaped structure” conformation was found in Ank^GAG^1D4_NC-NC_ and Ank^GAG^1D4_NC-CN_ (Figure 3E,K). Conversely, the opposite direction of the binding domain was observed for Ank^GAG^1D4_NC-NC_.

### 2.3. Structural Analysis Showed Functional Binding Property of Ank^GAG^1D4 Dimers

Owing to the vdw calculation of the interactive energy generated from two CAp24s, the possibility of ankyrin dimers simultaneously capturing with CAp24s was validated. Slightly over half (5.6%) of the Ank^GAG^1D4_NC-NC_ conformations enabled the simultaneous binding of two CAp24s (Table 1 and Figure 3B,F). In contrast, 44.9% showed overlapping coordinates, resulting in the collision of two CAp24 structures or the hindrance of the binding surface of another module from the previously bound CAp24 (Figure 3D). These findings suggest a 1:1 ratio of Ank^GAG^1D4_NC-NC_ to CAp24. Since 88.6% of the Ank^GAG^1D4_NC-CN_ conformation demonstrated accessible coordinates, the simultaneous accommodation of two CAp24s was more feasible (Figure 3H,J). Only 11.4% of Ank^GAG^1D4_NC-CN_ presented a 1:1 interaction.

### 2.4. Bifunctional Module Relies on Dimeric Ank^GAG^1D4_NC-CN_

**Figure 4:**
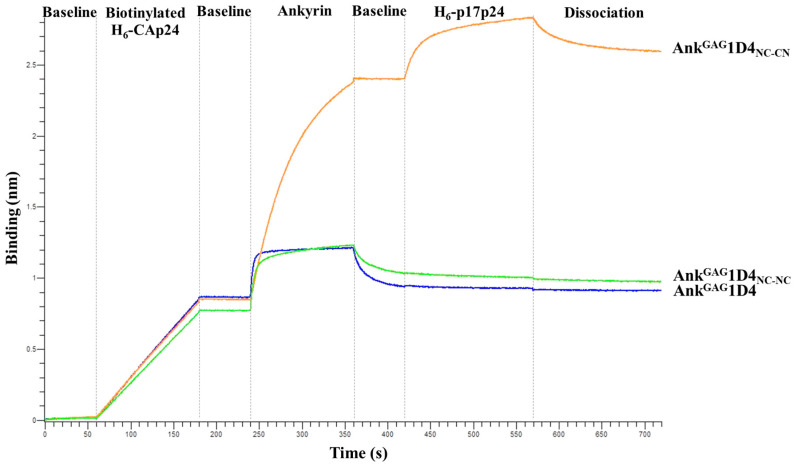
Binding characteristics of monomeric Ank^GAG^1D4, dimeric Ank^GAG^1D4_NC-NC_, and Ank^GAG^1D4_NC-CN_. Ligand binding of monomeric and dimeric Ank^GAG^1D4 is demonstrated by a sandwich assay using bio-layer interferometry. The biotinylated H_6_-CAp24 was immobilized to the Octet streptavidin biosensors and sequentially probed with the monomeric or dimeric ankyrins. Subsequently, H_6_-p17p24 was loaded to monitor the secondary interaction signal. This sensorgram is representative of the triplicate experiment.

The results above supported the distinctive binding activity of Ank^GAG^1D4_NC-NC_ and Ank^GAG^1D4_NC-CN_ analyzed by BLI. The functional modules of monomeric Ank^GAG^1D4, Ank^GAG^1D4_NC-NC_, and Ank^GAG^1D4_NC-CN_ were assessed by BLI with two CAp24 molecules (Figure 4). The biotinylated H_6_-CAp24 was loaded on the biosensor, followed sequentially by binding with the monomeric, dimeric Ank^GAG^1D4_NC-NC_ or Ank^GAG^1D4_NC-CN_ and then the H_6_-p17p24. Ank^GAG^1D4_NC-CN_ bound both CAp24 molecules simultaneously, whereas the monomeric Ank^GAG^1D4 and Ank^GAG^1D4_NC-NC_ could not bind the CAp24 using the second arm (Figure 4A). Ank^GAG^1D4_NC-NC_ is very fast off-rate comparable to monomer, indicating weak binder. Corresponding to binding kinetic constants, Ank^GAG^1D4_NC-CN_ (K_D_ < 1.0 × 10^−12^ M) is significantly stronger than Ank^GAG^1D4_NC-NC_ (K_D_ = 1.9 × 10^−8^ M) (Table 2). These data confirmed that both binding pockets of the dimeric Ank^GAG^1D4_NC-CN_ were active, while Ank^GAG^1D4_NC-NC_ has only one main binding site with a much weaker second binding site.

### 2.5. Ankyrin Expression Is Shunted to the Plasma Membrane and Does Not Interfere CD4 Expression in SupT1 Cells

**Figure 5:**
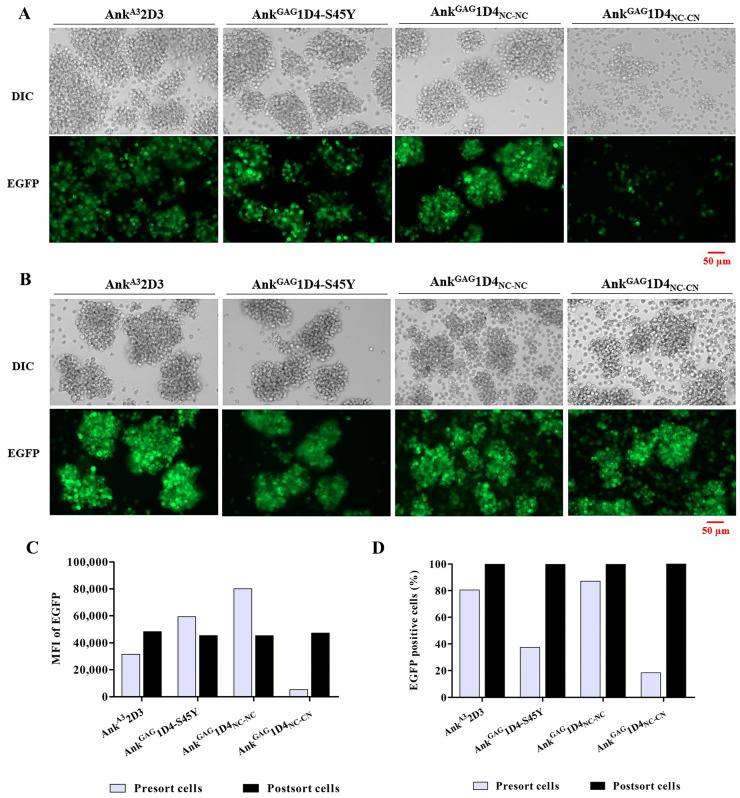
Expression of ankyrin-EGFP in transduced SupT1 cells: (**A**) At 48 h post-transduction, SupT1 cells showed EGFP expression under inverted fluorescence microscopy. These cells were sorted for an equivalent in ankyrin expression; (**B**) Post-sort cells showed EGFP expression under inverted fluorescence microscopy. Cell imaging was performed at 20× magnificent using Zeiss Colibri 7. Scale bar: 50 µm.; (**C**) The MFI of EGFP; and (**D**) percentage of EGFP positive cells of transduced SupT1 cells was determined using flow cytometry. Ank^A3^2D3, Ank^GAG^1D4-S45Y, Ank^GAG^1D4_NC-NC_ and Ank^GAG^1D4_NC-CN_ represent SupT1 cells expressing Myr (+) Ank^A3^2D3-EGFP, Myr (+) Ank^GAG^1D4-S45Y-EGFP, Myr (+) Ank^GAG^1D4_NC-NC_-EGFP, and Myr (+) Ank^GAG^1D4_NC-CN_-EGFP, respectively.

According to an in vitro study in bifunctional modules and molecular structure, dimeric ankyrins are promising in improved anti-HIV-1 activity. Thus, intracellular anti-HIV-1 activity was observed in SupT1 cells. SupT1 cells were transduced with VSV-G pseudotyped lentiviral vector, which carries a gene that encodes myristoylated ankyrin with an enhanced green fluorescent protein (EGFP), a fluorescence tag protein. Ankyrins in this study includes Myr (+) Ank^A3^2D3, Myr (+) Ank^GAG^1D4-S45Y, Myr (+) Ank^GAG^1D4_NC-NC_, and Myr (+) Ank^GAG^1D4_NC-CN_. At 48 h post-transduction, EGFP expression in SupT1 cells was determined by fluorescence microscopy. Each of transduced SupT1 cells showed EGFP expression, indicating an ankyrin protein expression in cells (Figure 5A). Each ankyrin-expressing cell was sorted at the same EGFP intensity to obtain an equivalence in ankyrin expression. After cell sorting, all ankyrin-expressing SupT1 cells showed EGFP expression under fluorescence microscopy (Figure 5B). The level of ankyrin expression was further determined by flow cytometry. The mean fluorescence intensity (MFI) of ankyrin-EGFP in SupT1 cells suggested a comparable ankyrin expression in SupT1 cells. MFI of EGFP was 4.83 × 10^4^, 4.53 × 10^4^, 4.53 × 10^4^, and 4.71 × 10^4^ in Ank^A3^2D3, Ank^GAG^1D4-S45Y, Ank^GAG^1D4_NC-NC_, and Ank^GAG^1D4_NC-CN_ expressing cell (Figure 5C). Additionally, the percentage of EGPF positive cells in post-sort Ank^A3^2D3, Ank^GAG^1D4-S45Y, Ank^GAG^1D4_NC-NC_, and Ank^GAG^1D4_NC-CN_ expressing cells was 100, 99.8, 99.8, and 99.8%, respectively (Figure 5D).

**Figure 6:**
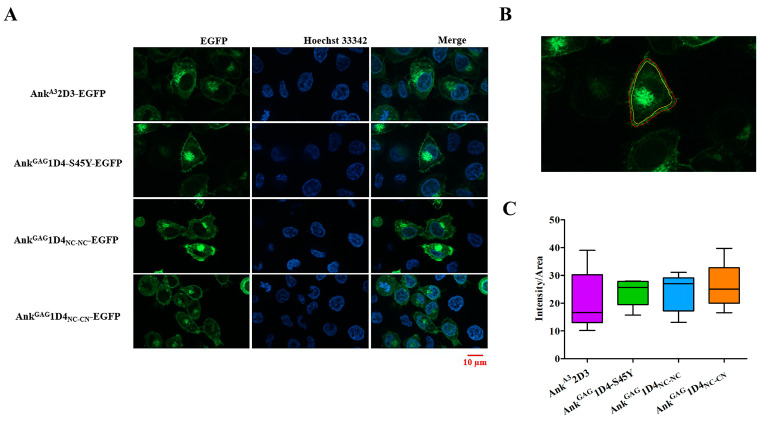
Ankyrin localization in HeLa cells. At 48 h post-transduction, ankyrin-expressing HeLa cells were stained with Hoechst 33342: (**A**) ankyrin localization in HeLa cells was determined under Zeiss Colibri 7 at 100× magnification with Apotome. Green represents ankyrin-EGFP expression, and blue indicates the nucleus of HeLa cells; (**B**) EGFP intensity in a ring was determined by subtracting the intensity of the outer ring (Red line) and inner ring (yellow line); (**C**) The EGFP intensity per area (Intensity/Area) in each ankyrin-expressing HeLa cell was calculated (*n* = 5) as follows: (outer ring intensity-inner ring intensity)/(outer ring area-inner ring area). Scale bar: 10 µm. Ank^A3^2D3, Ank^GAG^1D4-S45Y, Ank^GAG^1D4_NC-NC_ and Ank^GAG^1D4_NC-CN_ represent SupT1 cells expressing Myr (+) Ank^A3^2D3-EGFP, Myr (+) Ank^GAG^1D4-S45Y-EGFP, Myr (+) Ank^GAG^1D4_NC-NC_-EGFP, and Myr (+) Ank^GAG^1D4_NC-CN_-EGFP, respectively.

Additionally, Ankyrin localization was also determined in HeLa cells by apotome imaging. Ankyrin showed a predominant expression at the plasma membrane and lower distribution in the cytoplasm (Figure 6A). Apotome imaging suggested that ankyrins were shunted to the plasma membrane, which supports the possibility of ankyrin inhibiting viral replication. Additionally, ankyrin distribution at the plasma membrane was not different in each ankyrin-expressing cell (Figure 6B).

**Figure 7:**
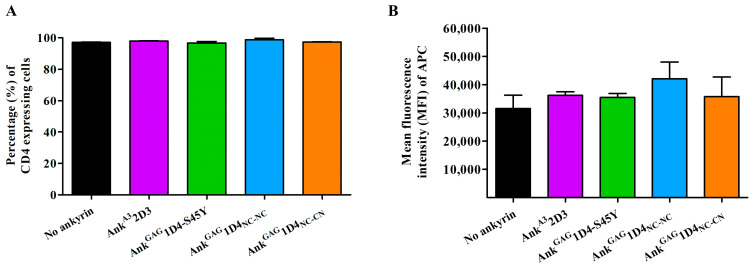
Surface CD4 expression in ankyrin-expressing SupT1 cells. SupT1 and ankyrin-expressing SupT1 cells were stained with APC-conjugated anti-human CD4 antibody: (**A**) Percentage of CD4 positive cells; and (**B**) The MFI of APC was determined by flow cytometry. The data represent the mean ± SD of the triplicate experiment. Ank^A3^2D3, Ank^GAG^1D4-S45Y, Ank^GAG^1D4_NC-NC_ and Ank^GAG^1D4_NC-CN_ represent SupT1 cells expressing Myr (+) Ank^A3^2D3-EGFP, Myr (+) Ank^GAG^1D4-S45Y-EGFP, Myr (+) Ank^GAG^1D4_NC-NC_-EGFP, and Myr (+) Ank^GAG^1D4_NC-CN_-EGFP, respectively.

As a consequence of the N-terminus myristoylated signal, ankyrins were targeted to the inner leaflet of the plasma membrane. Since CD4 is a crucial receptor for HIV-1 entry, it is necessary to determine whether an expression of ankyrin interfered with surface CD4 expression in SupT1 cells. Flow cytometry showed the number of CD4-positive cells in ankyrin-expressing SupT1 to no ankyrin cells (Figure 7A). MFI of CD4 in SupT1 cells and ankyrin-expressing SupT1 cells (Myr (+) Ank^A3^2D3 and Myr (+) Ank^GAG^1D4-S45Y, Myr (+) Ank^GAG^1D4_NC-NC_, and Myr (+) Ank^GAG^1D4_NC-CN_) was 3.16 × 10^4^, 3.62 × 10^4^, 3.56 × 10^4^, 4.21 × 10^4^, and 3.57 × 10^4^, respectively (Figure 7B). These data suggest that myristoylated-ankyrin expression in SupT1 did not interfere with CD4 contents on the cell surface of SupT1 cells.

### 2.6. Dimeric Ankyrins Improves Antiviral Activity Than Monomeric Ankyrins against HIV-1 Replication

**Figure 8:**
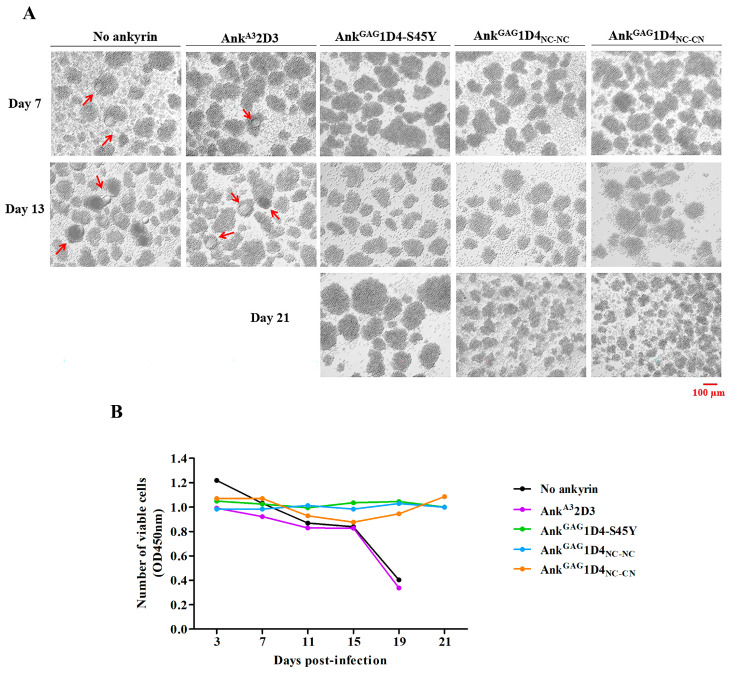
Cell morphology and cell viability of HIV-1 NL4-3 WT infected SupT1 cells. SupT1 and ankyrin-expressing SupT1 cells were infected with 10 MOI of HIV-1 NL4-3 WT virus. (**A**) Cell morphology was observed under inverted microscopy. Cell imaging was performed at 10× magnification using Zeiss Axio vert A1. The image shown in this figure is representative of several image fields. Scale bar: 100 µm. The arrows point to syncytium cells which are observed in infected cells. (**B**) Cell viability of infected cells was determined by CCK-8 assay. The data represent the mean from triplicate wells. Ank^A3^2D3, Ank^GAG^1D4-S45Y, Ank^GAG^1D4_NC-NC_ and Ank^GAG^1D4_NC-CN_ represent SupT1 cells expressing Myr (+) Ank^A3^2D3-EGFP, Myr (+) Ank^GAG^1D4-S45Y-EGFP, Myr (+) Ank^GAG^1D4_NC-NC_-EGFP, and Myr (+) Ank^GAG^1D4_NC-CN_-EGFP, respectively.

The intracellular anti-HIV-1 activity of dimeric ankyrins was determined in HIV-1 NL4-3 infected SupT1 cells. In this experiment, SupT1 and Ank^A3^2D3 expressing SupT1 served as no ankyrin control and irrelevant ankyrin control, respectively. At 7 days post-infection, syncytium cells were observed in SupT1 and Ank^A3^2D3 expressing SupT1 (Figure 8A). On the contrary, Ank^GAG^1D4-S45Y, Ank^GAG^1D4_NC-NC_, and Ank^GAG^1D4_NC-CN_ expressing cells show normal cell morphology until 21 days post-infection. Additionally, the cell viability of infected cells was monitored by CCK-8 assay. SupT1 cell and Ank^A3^2D3 expressing SupT1 cells showed a dramatically decreased cell viability at day 19 post-infection (Figure 8B). The result of SupT1 and Ank^A3^2D3 representing cell viability is relevant to cell morphology. In contrast to other ankyrin-expressing cells, cell viability was extended to 21 days.

**Figure 9:**
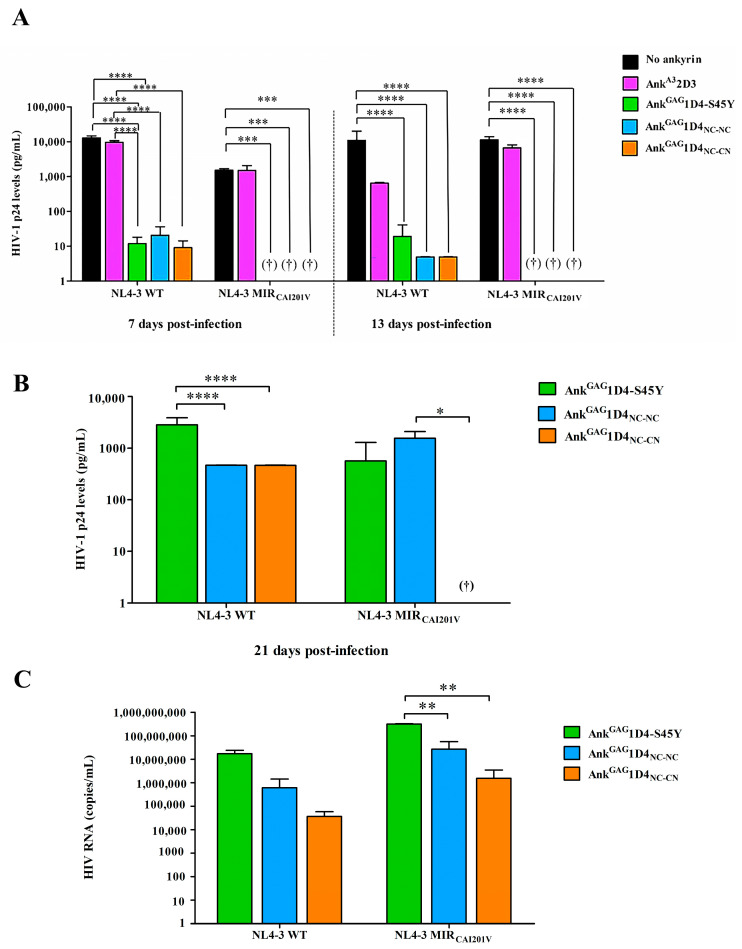
HIV-1 production in infected SupT1 and ankyrin-expressing SupT1 cells. Culture supernatant of HIV-1 WT or HIV-1 NL4-3 MIR_CAI201V_ infected cells was collected at (**A**) 7, 13, and (**B**) 21 days post-infection to determine the level of HIV-1 p24 by ELISA. “†” represents an undetectable of HIV-1 CAp24 level. (**C**) HIV RNA in the day 21 culture supernatant was evaluated by real-time RT-qPCR. Data represent mean ± SD from duplicate wells. * *p* ≤ 0.05, ** *p* ≤ 0.01, *** *p* ≤ 0.001, **** *p* ≤ 0.0001 using one-way ANOVA. NL4-3 WT and NL4-3 MIR_CAI201_ indicated the infection of cells with HIV-1 NL4-3 WT and HIV-1 NL4-3 MIR_CAI201V_ virus. Ank^A3^2D3, Ank^GAG^1D4-S45Y, Ank^GAG^1D4_NC-NC_ and Ank^GAG^1D4_NC-CN_ represent SupT1 cells expressing Myr (+) Ank^A3^2D3-EGFP, Myr (+) Ank^GAG^1D4-S45Y-EGFP, Myr (+) Ank^GAG^1D4_NC-NC_-EGFP, and Myr (+) Ank^GAG^1D4_NC-CN_-EGFP, respectively.

HIV-1 production in HIV-1 infected SupT1 cells and ankyrin-expressing SupT1 cells was evaluated using HIV-1 p24 ELISA. During culture, the culture supernatant of 7, 13, and 21 days post-infection was harvested and tested for HIV-1 CAp24 level by ELISA. At 7 days post-infection, the level of HIV-1 p24 in the culture supernatant of SupT1 and Ank^A3^2D3-expressing SupT1 cells was detected at 1.39 × 10^4^ and 1.02 × 10^4^ pg/mL (Figure 9A). Whereas, Ank^GAG^1D4-S45Y, Ank^GAG^1D4_NC-NC_, and Ank^GAG^1D4_NC-CN_ showed a higher potency to suppress HIV-1 propagation. Although Ank^GAG^1D4-S45Y suppressed HIV-1 release, their single functional module loss inhibitory activity as a higher CAp24 level was detected on day 21 compared to dimeric ankyrins (Figure 9B). Interestingly, Ank^GAG^1D4_NC-CN_ showed the highest potency in HIV-1 inhibition. HIV-1 virion packaging was determined as an HIV RNA copy by real-time RT-qPCR. An HIV RNA copy number in Ank^GAG^1D4-S45Y, Ank^GAG^1D4_NC-NC_, and Ank^GAG^1D4_NC-CN_ expressing cells was 1.76 × 10^7^, 6.14 × 10^5^ and 3.69 × 10^4^ copies/mL, respectively (Figure 9C). Corresponding to previous findings, the specific interaction of ankyrin to HIV-1 CAp24 interferes with the assembly process leading to a decrease in virion release and disturbing the viral RNA packaging [5]. This finding confers the improvement of anti-HIV-1 activity against HIV-1 NL4-3 WT replication in dimeric ankyrins.

### 2.7. Dimeric Ankyrin Provides Superior Anti-Viral Activity against HIV-1 MIR Virus

**Figure 10:**
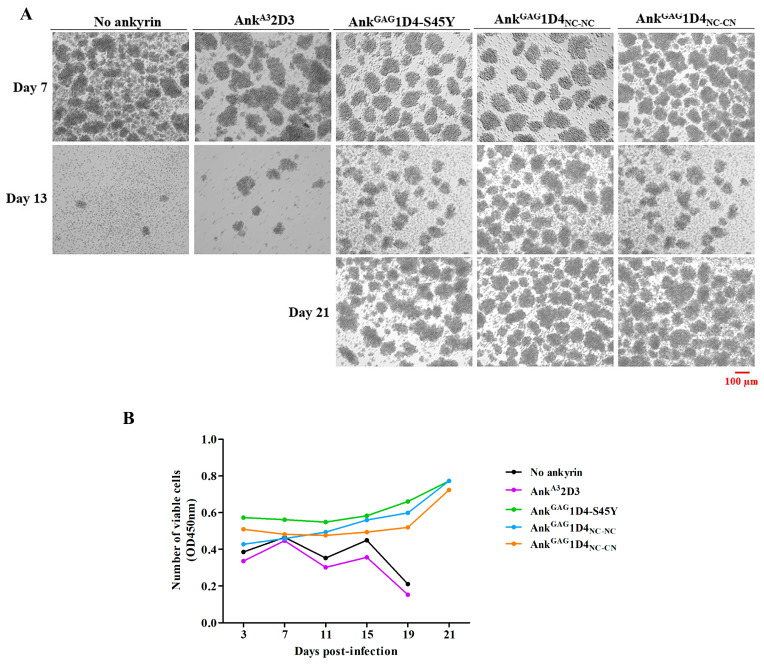
Cell morphology and cell viability of HIV-1 NL4-3 MIR_CAI201V_ infected SupT1 cells. SupT1 and ankyrin-expressing SupT1 cells were infected with 10 MOI of HIV-1 NL4-3 MIR_CAI201V_ virus. (**A**) Cell morphology was observed under inverted microscopy. Cell imaging was performed at 10× magnification using Zeiss Axio vert A1. The image shown in this figure is representative of several image fields. Scale bar: 100 µm(**B**) Cell viability of infected cells was determined by CCK-8 assay. The data represent the mean from triplicate wells. Ank^A3^2D3, Ank^GAG^1D4-S45Y, Ank^GAG^1D4_NC-NC_ and Ank^GAG^1D4_NC-CN_ represent SupT1 cells expressing Myr (+) Ank^A3^2D3-EGFP, Myr (+) Ank^GAG^1D4-S45Y-EGFP, Myr (+) Ank^GAG^1D4_NC-NC_-EGFP, and Myr (+) Ank^GAG^1D4_NC-CN_-EGFP, respectively.

The anti-HIV-1 potency of dimeric ankyrins was further investigated in the HIV-1 CA-mutation virus. In this study, HIV-1 NL4-3 MIR_CAI201V_ was used as a CA-mutation model. According to cell morphology, no syncytia was observed in HIV-1 NL4-3 MIR_CAI201V_-infected cells. However, at day 13 post-infection, SupT1, and Ank^A3^2D3-expressing SupT1 cells show a clumping appearance and cell death (Figure 10A). SupT1 and Ank^A3^2D3-expressing SupT1 cells show unstable cell viability and tremendously decrease on day 15 post-infection (Figure 10B).

At 7 days post-infection, the CAp24 level in infected SupT1 and Ank^A3^2D3 expressing SupT1 cells was detected at 1.58 × 10^3^ and 1.80 × 10^3^ pg/mL (Figure 9A). In contrast to other ankyrins, Ank^GAG^1D4-S45Y, Ank^GAG^1D4_NC-NC_, and Ank^GAG^1D4_NC-CN_ exhibited a long-lasting anti-HIV-1 activity as undetectable in CAp24 levels. Nonetheless, this CA-mutation is slow propagation than the WT strain [16,18]. Although CAp24 levels increased in 21 days post-infection, dimeric Ank^GAG^1D4_NC-NC_ and Ank^GAG^1D4_NC-CN_ showed a significant suppression compared with Ank^GAG^1D4-S45Y (Figure 9B). Interestingly, Ank^GAG^1D4_NC-CN_ showed the highest efficiency in inhibiting HIV-1 replication. Real-time RT-qPCR confirmed a higher potency of Ank^GAG^1D4_NC-CN_ due to the lowest HIV-1 RNA copy (Figure 9C). Although the CAp24 level in Ank^GAG^1D4-S45Y expressing cells was slightly different from dimeric ankyrin, RNA copy showed a significantly higher degree than in dimeric ankyrins expressing cells. This data suggested a possibility of aberrant viral core formation, which defects in the viral infectivity of the virions [19]. These results suggested the superior activity of Ank^GAG^1D4_NC-CN_ to inhibit the multiplication of HIV-1 NL4-3 MIR_CAI201V_ virus.

## 3. Discussion

Designed ankyrin repeat proteins (DARPins) have been selected and focused on therapeutic application. DARPin MP0274, targeting human epidermal growth factor 2 (HER2), has been evaluated in clinical trials for cancer treatment [20]. Improving the binding affinity of DARPins could enhance antiviral activity. Trimeric DARPin, Ensovibep, was explicitly designed to inactivate severe acute respiratory syndrome coronavirus 2 (SARS-CoV-2). It includes three individual DARPin domains neutralizing against trimeric SARS-CoV-2 spike protein to reduce viral replication in the lower and upper respiratory tract [21]. Ank^GAG^1D4-S45Y mutant as a specific CAp24 domain inhibitor of HIV-1 Gag was constructed using sited-direct mutagenesis. The binding affinity of Ank^GAG^1D4-S45Y mutant (KD = 45 nM) was partly enhanced and inhibited either HIV-1 wild-type or the HIV maturation inhibitor-resistant strain for intracellular activity compared to parental Ank^GAG^1D4 (KD = 109 nM) [6,7]. Dimeric Ank^GAG^1D4 was generated to further improve its binding activity and evaluated for the binding activity [11] (Figure S1). The (G_4_S)_4_ linker was applied to the dimeric ankyrin structure, providing flexibility and high solubility of molecules [22]. The G_4_S linker provides flexibility and solubility in bidomain molecules, such as scFSH scaffolds [23], and αRep homo-bidomain (A3_A3) [24]. For these reasons, the (G_4_S)_4_ linker might support the intracellular activity of dimeric ankyrin.

Regarding the BLI and MD, Ank^GAG^1D4_NC-NC_ and Ank^GAG^1D4_NC-CN_ show different binding characteristics. Bio-layer interferometry demonstrated that Ank^GAG^1D4_NC-CN_ remarkably promoted the binding activity, achieving the pM avidity characteristic. The secondary interaction signal of Ank^GAG^1D4_NC-CN_ in BLI suggested the bifunctional motif in contrast to Ank^GAG^1D4_NC-NC_. In contrast, the binding activity of Ank^GAG^1D4_NC-NC_ was insignificantly enhanced and lacked the ability to capture the additional p17p24. This suggests the steric hindrance introduced after interacting with the immobilized p24, causing improper orientation of the Ank^GAG^1D4_NC-NC_ that limits the access of p17p24 to the second binding site. This data consensus with the MD simulation in which the binding surfaces of Ank^GAG^1D4_NC-CN_ synchronically interact with CAp24. Half of the Ank^GAG^1D4_NC-NC_ conformations preferred a 1:1 interaction, whereas most Ank^GAG^1D4_NC-CN_ favored a 1:2 interaction. This phenomenon confers the potential of Ank^GAG^1D4_NC-CN_ to interfere with HIV-1 Gag-assemble compared to Ank^GAG^1D4_NC-NC_ and monomeric Ank^GAG^1D4. Previously, an anti-HIV-1 production of Ank^GAG^1D4-S45Y has been expressed, and the leakage of HIV-1 virions production was detected in the late infection [7]. Although Ank^GAG^1D4-S45Y enhanced anti-HIV-1 activity, both dimeric ankyrins were superior. Additionally, the anti-HIV-1 activity of dimeric ankyrin was also investigated in the HIV-1 NL4-3 MIR_CAI201V_ virus, which served as a CA-mutation virus model. Since this mutation occurs in CA-CTD, the monomeric and dimeric forms of Ank^GAG^1D4 were presumed to inhibit HIV-1 NL4-3 MIR_CAI201V_ virus replication. In this study, Ank^GAG^1D4-S45Y and dimeric ankyrins showed a remarkable reduction in the viral progeny production in HIV-1 NL4-3 MIR_CAI201V_ infection. This finding suggested that Ank^GAG^1D4_NC-NC_ and Ank^GAG^1D4_NC-CN_ retain their functionality to accommodate different CAp24 molecules promising to interfere with Gag packaging simultaneously. Interestingly, the bifunctional property of Ank^GAG^1D4_NC-NC_ was not indicated by BLI, whereas the inhibitory effect was significantly improved in comparison with Ank^GAG^1D4-S45Y. We hypothesized that the intercellular flexibility of Ank^GAG^1D4_NC-NC_ is more compromised than in vitro. In addition, the dimension of Ank^GAG^1D4_NC-NC_ is doubling of Ank^GAG^1D4-S45Y, thus more efficient in perturbing capsid assembly. Further exploration should be performed to explain this phenomenon.

The results of this study suggested that the inverted second module of Ank^GAG^1D4_NC-CN_ efficiently conforms to bifunctional activity. Considering the conformational structure of Ank^GAG^1D4_NC-CN_, it conducts a superior inhibitory response than Ank^GAG^1D4_NC-NC_. To provide the occupied dimension for CAp24, it is crucial to consider the effect of the extended distance between the binding surfaces apart from the flexibility of the linker. When dimeric ankyrins are expressed in the infected cells, they enhance the inhibitory activity against the HIV-1 NL4-3 WT virus. On days 7 and 13, the difference of CAp24 in the presence of Ank^GAG^1D4-S45Y and dimers is significant compared to no ankyrin control (>1000-fold). However, after day 19 the control groups were terminated since the cell viability drastically decreased. Thus, on day 21 the CAp24 level was determined only with Ank^GAG^1D4-S45Y and dimers. Most infected cells harboring Ank^GAG^1D4-S45Y and dimers survived and continuously generated a small number of viral particles. Although the fold difference of CAp24 among this group is not much, the protection of dimers was significantly different from Ank^GAG^1D4-S45Y in the WT virus. Moreover, dimeric ankyrins retain their viral protection activity against HIV-1 NL4-3 MIR_CAI201V_ virus replication in SupT1 cells. Other drug-resistant strains regarding mutation at the capsid domain will be further evaluated. A substantial increase in pretreatment drug resistance prevalence indicated that first-line ART could not completely eradicate HIV-1 infection [25]. Based on our findings, gene therapy using Ank^GAG^1D4_NC-CN_ could be feasible to integrate with highly active antiretroviral therapy (HAART) for sustainable treatment.

Dimeric ankyrin Ank^GAG^1D4_NC-CN_ provides the avidity function regarding the flexible structure and the simultaneous accessibility of the interacting modules. The advantage of Ank^GAG^1D4_NC-CN_ is the forceful interaction against the juxtaposed Gag molecules, thus efficiently interfering with the HIV-1 assembly process.

## 4. Materials and Methods

### 4.1. Construction of Inverted Ank^GAG^1D4

The amino acid sequence of the inverted Ank^GAG^1D4 is a backwardly read protein sequence of Ank^GAG^1D4 (PDB ID: 4HLL) [26], which is used for the initial coordinates of the inversion. In the PDB file format, the atom serial number of the parent coordinates was rearranged in a mirror image order. The backbone atom names were subsequently modified. Two oxygens of the carboxyl group were removed, and the carbon atom of this group was swapped with the nitrogen atom of the amino group. The modified coordinate structure was eventually used as a template for the three-dimensional structure of the inverted Ank^GAG^1D4. The structural model was constructed using the user-template mode of SWISS-MODEL software [27].

### 4.2. Structural Modeling of Ank^GAG^1D4 Dimer

The initial three-dimensional model for the dimers was generated from the first ankyrin module 1D4 and connected to a flexible linker, Ank^GAG^1D4-linker. The (G_4_S)_4_ linker was extracted from the crystal structure of scFv-IL-1B (PDB ID: 2KH2) [28]. It was inserted with residues LLQ at the N-terminus and with residues TSDL at the C-terminus. The residues LLQ and TSDL were superimposed with the N-terminal and C-terminal amino acids of the first and second ankyrin modules, respectively. After superimposing the LL residues of Ank^GAG^1D4 with the LL residues of the linker, two possible conformations of the Ank^GAG^1D4-linker were generated. Atomistic molecular dynamics simulations of each Ank^GAG^1D4-linker were performed using NAMD software version 1.1422 for 5 ns under the CHARMM36 force field [29]. TIP3P59 water was employed at 310 K and 1 atm. The folding dynamics were simulated with an integration time step of 2 fs, evaluated nonbonded every 1 fs, and updated electrostatics every 2 fs. Afterward, the last four residues (TSDL) of the MD Ank^GAG^1D4-linker were superimposed with another Ank^GAG^1D4 or an inverted Ank^GAG^1D4 module to create the dimeric Ank^GAG^1D4_NC-NC_ and Ank^GAG^1D4_NC-CN_, respectively. The dimers depicting the crashing structures of the ankyrin modules and linker were excluded. The remaining generated structures were analyzed for dimer behavior in interacting with CAp24. The duplex ankyrin modules were conclusively superimposed with the docking complex of Ank^GAG^1D4 and CAp24 from a previous study [19]. The binding ratio of each dimer to CAp24 was also investigated.

### 4.3. Determination of the Distance of Binding Sites

The distance between the binding sites was measured between two carbon alpha atoms at L53 of the first and second modules of the Ank^GAG^1D4_NC-NC_ dimer. The Ank^GAG^1D4_NC-CN_ binding distance was determined from L53 of the first module to the reversed L53 residue in the second inverted module. The L53 of Ank^GAG^1D4 was in the middle of S45 and Y56, which was used to confirm critical residues interacting with CAp24. The distance was calculated using NAMD software [30].

### 4.4. Analysis of the Binding Ratio of Ankyrin Dimer to CAp24

The geometry of CAp24 molecules extracted from the generated structures in the ‘structural modeling of Ank^GAG^1D4 dimer’ described above was deployed to obtain the van der Waal (vdw) interactive energy using NAMD software. Suppose the vdw energy was ≤0, the 1:2 interaction without colliding with each captured CAp24 in binding with dimers was acceptable. In contrast, the positive vdw value reflected a 1:1 interaction in which two CAp24 were not simultaneously captured.

### 4.5. Binding Activity of Dimeric Ank^GAG^1D4

Bio-layer interferometry (BLI) was performed to analyze the binding activity of dimeric Ank^GAG^1D4 on an Octet K2 system (ForteBio, Menlo Park, CA, USA) in a sandwich format. Streptavidin biosensors were loaded with biotinylated H_6_-CAp24 (2 µg/mL for 60 s), dipped in the dimeric ankyrins (10 µg/mL for 120 s) compared with monomer (2.5 µg/mL for 120 s), then probed with an H_6_-p17p24 (10 µg/mL for 150 s). Baselines were established before and after the loading step. All assays were performed in 2% BSA in PBST (PBS containing 0.05% Tween 20). Binding curves were fit to a 1:1 binding model on Octet data analysis software version 9.0.

### 4.6. Cell Lines and Plasmids

SupT1 cells (ATCC) were cultured in Roswell Park Memorial Institute (RPMI) 1640 medium (Gibco) supplemented with 10% heat-inactivated fetal bovine serum (HI-FBS) (Gibco), 100 U/mL of penicillin (Gibco), 100 µg/mL of streptomycin (Gibco), and 2 mM of L-glutamine (Gibco). HEK293T cells and HeLa cells (ATCC) were cultured in Dulbecco’s Modified Eagle Medium (DMEM) (Gibco) supplemented with 10% HI-FBS, 100 U/mL of penicillin, 100 µg/mL of streptomycin, and 2 mM of L-glutamine.

pNL4-3 plasmid, the infectious HIV-1 NL4-3 molecular clone (NIH), was used to produce the HIV-1 NL4-3 laboratory strain virus. pNL4-3 MIR_CAI201V_, the molecular clone of the HIV-1 MIR virus, was constructed as previously described [7].

CGW transfer vector was constructed, and a third-generation lentiviral vector was used as the backbone vector to transfer the genes for myristoylated ankyrins into target cells. In addition, this vector carried EGFP acts as a reporter gene.

### 4.7. Lentivirus Production

Lentiviral transfer plasmid, CGW-Myr (+) Ank^GAG^1D4-EGFP, was constructed and used as a backbone vector for generating the CGW-Myr (+) Ank^GAG^1D4_NC-NC_ -EGFP and CGW-Myr (+) Ank^GAG^1D4_NC-CN_-EGFP. The PCR products were treated with EcoRI and BstXI and cloned into the CGW vector using T4 DNA ligase (ThermoFisher Scientific, MA, USA). The ligation products were transformed into XL-1 blue competent *E. coli* cells for plasmid amplification. The transformed XL-1 blue cells were grown on Luria–Bertani (LB) agar supplemented with 100 µg/mL of ampicillin at 37 °C for 16 h. A single bacterial colony was picked and cultured in super broth (SB) supplemented with 100 µg/mL of ampicillin at 37 °C for 16 h. After culturing, the plasmid was extracted and purified using a DNA extraction kit (GeneAll Biotechnology, Seoul, Republic of Korea). DNA sequencing was performed to confirm the correct plasmid construction.

HEK293T cells were seeded on a 10-cm dish for 16 h. Cells were co-transfected with 10 µg of lentiviral transfer plasmid (including CGW-Myr (+) Ank^A3^2D3-EGFP, CGW-Myr (+) Ank^GAG^1D4-S45Y-EGFP, CGW-Myr (+) Ank^GAG^1D4_NC-NC_ -EGFP and CGW-Myr (+) Ank^GAG^1D4_NC-CN_ -EGFP), 6.5 µg of pMDLg/pRRE, 2.5 μg of pRSV-Rev, and 3.5 μg of pMD.2G using MirusTransIT-X2 Dynamic Delivery System (Mirus Bio, Madison, WI, USA). Viral particles were harvested from culture supernatant collected at 48 h post-transfection. Lentiviral vector titer was determined by transduction in HEK293T cells and observed EGFP positive cells under fluorescent microscopy and flow cytometry.

### 4.8. Ankyrin-Expressing SupT1 Cells Establishment

SupT1 cells were transduced with VSV-G pseudotyped lentiviral vector at a multiplicity of infection (MOI) of 1, with 8 ug/mL of polybrene. VSV-G pseudotyped lentiviral vector used in this experiment included VSVG–CGW–Myr (+) Ank^GAG^2D3-EGFP, VSVG–CGW–Myr (+) Ank^GAG^1D4-S45Y-EGFP, VSVG–CGW–Myr (+) Ank^GAG^1D4_NC-NC_-EGFP and VSVG–CGW–Myr (+) Ank^GAG^1D4_NC-CN_-EGFP. These cells were spinoculated at 2500× *g* for 1.30 h and cultured for 16 h. After incubation, these cells were washed and cultured in a 10% HI-FBS-RPMI 1640 medium. Ankyrin-EGFP expression in cells was observed under an inverted fluorescence microscope. The percentage of EGFP-positive cells was determined by BD Accuri^TM^C6 (BD biosciences, Franklin Lakes, NJ, USA). SupT1 stable cells were sorted by a BD FACSMelody^TM^ cell sorter (BD Biosciences, Franklin Lakes, NJ, USA) to obtain the comparable expression level of ankyrin.

### 4.9. Ankyrin Subcellular Localization

HeLa cells were transduced with VSV-G pseudotyped lentiviral vector at an MOI of 1, with 8 ug/mL of polybrene. VSV-G pseudotyped lentiviral vector carries ankyrins gene as mentioned above. After 48 h post-transduction, transduced cells were seeded on a glass coverslip overnight. Cells were incubated with Hoechst 33342 (ThermoFisher Scientific, Waltham, MA, USA) for nuclear staining. After washing, cells were fixed in 4% paraformaldehyde and mounted on a glass slide. Ankyrin localization was determined under fluorescent microscopy with apotome (100× magnification) using Zeiss Colibri 7. The mean fluorescent intensity was measured by ImageJ software (NIH). To compare the level of ankyrin expression at the plasma membrane, EGFP intensity, and ring area in ankyrin-expressing HeLa cells were calculated to intensity per area as follows: (outer ring intensity-inner ring intensity)/(outer ring area-inner ring area).

### 4.10. HIV-1 NL4-3 WT and HIV-1 NL4-3 MIRCAI201V Production

HIV-1 NL4-3 WT and HIV-1 NL4-3 MIR_CAI201V_ were produced as previously described [7]. Briefly, HEK293T cells were transfected with pNL4-3 WT or pNL4-3 MIR_CAI201V_ plasmid using MirusTransIT-X2 Dynamic Delivery System. At 48 h post-transfection, culture supernatant was harvested. The virions-containing culture supernatant was centrifuged at 335× *g* for 5 min and passed through a 0.45 µm filter membrane to remove the insoluble particles. The viral stock was aliquoted and kept at −80 °C. HIV-1 viral titer was determined using real-time reverse transcription quantification polymerase chain reaction (RT-qPCR) using COBAS AmpliPrep/COBAS TaqMan HIV-1 test.

### 4.11. HIV-1 Infection

SupT1 cells (no ankyrin) and ankyrin-expressing SupT1 cells were inoculated with 10 MOI of HIV-1 NL4-3 WT or HIV-1 NL4-3 MIR_CAI201V_ virus. In this experiment, SupT1 and Ank^GAG^2D3-expressing SupT1 cells served as no protection control. After 16 h incubation, these cells were washed 3 times. Cells were cultured in RPMI 1640 medium supplemented with 10% HI-FBS and subcultured every 2 days. During culture, cell morphology was observed under an inverted microscope. Additionally, cell viability was monitored using CCK-8 assay. Briefly, 100 µL of cells were added to a 96-well plate and incubated with 10 µL of CCK-8 solution (Abbkine, Wuhan, China). Following 30 min incubation, viable cells were monitored at 450 nm by a CLARIOstar microplate reader (BMG Labtech, Baden-Württemberg, Germany).

To determine viral release in infected cells, culture supernatants at 7, 13, and 21 days post-infection were harvested for ELISA and real-time RT-qPCR as described below.

### 4.12. Viral Release Monitoring

The concentration of HIV-1 CAp24 in the culture supernatant was evaluated using Genscreen^TM^ Ultra HIV-1 p24 ELISA kit (ELISA) (Bio-Rad, FR). The harvested culture supernatant was centrifuged to remove cell debris and unwanted particles. The viral particles in the culture supernatant were lysed with 1% Triton-X 100 before the assay. The absorbances at 450 nm were read using a microplate reader and calculated for HIV-1 CAp24 levels using the HIV-1 CAp24 standard curve. Additionally, HIV-1 production was monitored by quantifying HIV-1 RNA copy number in day 21 post-infection culture supernatant by real-time RT-qPCR using COBAS Ampliprep/COBAS TaqMan HIV-1 test (Roche, Basel, Switzerland).

### 4.13. Statistical Analysis

Data are presented as the mean ± SD from triplicate experiments. Statistical analysis was performed using one-way ANOVA. Statistical differences were considered significant at *p* ≤ 0.05 (indicated with asterisks).

## Figures and Tables

**Table 1:** Frequency of binding ratio of one dimer to CAp24 molecule(s).

	Binding Ratio of Dimer to CAp24 (*n* = 403)					
	Ank^GAG^1D4_NC-NC_			Ank^GAG^1D4_NC-CN_		
	1:0 ^1^	1:1 ^2^	1:2 ^3^	1:0 ^1^	1:1 ^2^	1:2 ^3^
number	6.0	181.0	216.0	0.0	46.0	357.0
%	1.5	44.9	53.6	0.0	11.4	88.6

^1^ Dimer cannot bind any CAp24; ^2^ Dimer enables binding to one CAp24; ^3^ Dimer enables binding to two CAp24s.

**Table 2:** Kinetic characterization of monomeric and dimeric Ank^GAG^1D4.

Variants	K_D_ (M)	K_on_ (M·s^−1^)	K_off_ (s^−1^)
Monomeric Ank^GAG^1D4	3.3 × 10^−8^	6.8 × 10^5^	2.2 × 10^−2^
Dimeric Ank^GAG^1D4_NC-NC_	1.9 × 10^−8^	1.3 × 10^5^	9.9 × 10^−3^
Dimeric Ank^GAG^1D4_NC-CN_	<1.0 × 10^−12^	1.1 × 10^5^	<1.0 × 10^−7^

## Data Availability

Data are contained within the article or Supplementary Materials.

## Supplementary Materials

The following supporting information can be downloaded at: https://www.mdpi.com/article/10.3390/ijms24065266/s1.

## References

[1] Schweizer A., Rusert P., Berlinger L., Ruprecht C.R., Mann A., Corthésy S., Turville S.G., Aravantinou M., Fischer M., Robbiani M. (2008). CD4-specific designed ankyrin repeat proteins are novel potent HIV entry inhibitors with unique characteristics. PLoS Pathog..

[2] Mann A., Friedrich N., Krarup A., Weber J., Stiegeler E., Dreier B., Pugach P., Robbiani M., Riedel T., Moehle K. (2013). Conformation-dependent recognition of HIV gp120 by designed ankyrin repeat proteins provides access to novel HIV entry inhibitors. J. Virol..

[3] Sakkhachornphop S., Hadpech S., Wisitponchai T., Panto C., Kantamala D., Utaipat U., Praparattanapan J., Kotarathitithum W., Taejaroenkul S., Yasamut U. (2018). Broad-Spectrum Antiviral Activity of an Ankyrin Repeat Protein on Viral Assembly against Chimeric NL4-3 Viruses Carrying Gag/PR Derived from Circulating Strains among Northern Thai Patients. Viruses.

[4] Nangola S., Urvoas A., Valerio-Lepiniec M., Khamaikawin W., Sakkhachornphop S., Hong S.-S., Boulanger P., Minard P., Tayapiwatana C.J.R. (2012). Antiviral activity of recombinant ankyrin targeted to the capsid domain of HIV-1 Gag polyprotein. Retrovirology.

[5] Moonmuang S., Maniratanachote R., Chetprayoon P., Sornsuwan K., Thongkum W., Chupradit K., Tayapiwatana C. (2022). Specific Interaction of DARPin with HIV-1 CA(NTD) Disturbs the Distribution of Gag, RNA Packaging, and Tetraspanin Remodelling in the Membrane. Viruses.

[6] Saoin S., Wisitponchai T., Intachai K., Chupradit K., Moonmuang S., Nangola S., Kitidee K., Fanhchaksai K., Lee V.S., Hong S.S. (2018). Deciphering critical amino acid residues to modify and enhance the binding affinity of ankyrin scaffold specific to capsid protein of human immunodeficiency virus type 1. Asian Pac. J. Allergy Immunol..

[7] Sornsuwan K., Thongkhum W., Pamonsupornwichit T., Carraway T.S., Soponpong S., Sakkhachornphop S., Tayapiwatana C., Yasamut U. (2021). Performance of Affinity-Improved DARPin Targeting HIV Capsid Domain in Interference of Viral Progeny Production. Biomolecules.

[8] Boersma Y.L., Chao G., Steiner D., Wittrup K.D., Plückthun A. (2011). Bispecific designed ankyrin repeat proteins (DARPins) targeting epidermal growth factor receptor inhibit A431 cell proliferation and receptor recycling. J. Biol. Chem..

[9] Chonira V., Kwon Y.D., Gorman J., Case J.B., Ku Z., Simeon R., Casner R.G., Harris D.R., Olia A.S., Stephens T. (2022). A potent and broad neutralization of SARS-CoV-2 variants of concern by DARPins. Nat. Chem. Biol..

[10] Rothenberger S., Hurdiss D.L., Walser M., Malvezzi F., Mayor J., Ryter S., Moreno H., Liechti N., Bosshart A., Iss C. (2022). The trispecific DARPin ensovibep inhibits diverse SARS-CoV-2 variants. Nat. Biotechnol..

[11] Juntit O.A., Yasamut U., Sakkhachornphop S., Chupradit K., Thongkum W., Srisawat C., Chokepaichitkool T., Kongtawelert P., Tayapiwatana C. (2022). Biological properties of reverse ankyrin engineered for dimer construction to enhance HIV-1 capsid interaction. Asian Pac. J. Allergy Immunol..

[12] Thenin-Houssier S., Valente S.T. (2016). HIV-1 Capsid Inhibitors as Antiretroviral Agents. Curr. HIV Res..

[13] Li G., Verheyen J., Rhee S.-Y., Voet A., Vandamme A.-M., Theys K. (2013). Functional conservation of HIV-1 Gag: Implications for rational drug design. Retrovirology.

[14] Martin D.E., Salzwedel K., Allaway G.P. (2008). Bevirimat: A Novel Maturation Inhibitor for the Treatment of HIV-1 Infection. Antivir. Chem. Chemother..

[15] Urano E., Ablan S.D., Mandt R., Pauly G.T., Sigano D.M., Schneider J.P., Martin D.E., Nitz T.J., Wild C.T., Freed E.O. (2016). Alkyl Amine Bevirimat Derivatives Are Potent and Broadly Active HIV-1 Maturation Inhibitors. Antimicrob. Agents Chemother..

[16] Waki K., Durell S.R., Soheilian F., Nagashima K., Butler S.L., Freed E.O. (2012). Structural and functional insights into the HIV-1 maturation inhibitor binding pocket. PLoS Pathog..

[17] Cevik M., Orkin C. (2019). Insights into HIV-1 capsid inhibitors in preclinical and early clinical development as antiretroviral agents. Expert Opin. Investig. Drugs.

[18] Urano E., Timilsina U., Kaplan J.A., Ablan S., Ghimire D., Pham P., Kuruppu N., Mandt R., Durell S.R., Nitz T.J. (2019). Resistance to Second-Generation HIV-1 Maturation Inhibitors. J. Virol..

[19] Welker R., Hohenberg H., Tessmer U., Huckhagel C., Kräusslich H.G. (2000). Biochemical and structural analysis of isolated mature cores of human immunodeficiency virus type 1. J. Virol..

[20] Vazquez-Lombardi R., Phan T.G., Zimmermann C., Lowe D., Jermutus L., Christ D. (2015). Challenges and opportunities for non-antibody scaffold drugs. Drug Discov. Today.

[21] Barkauskas C., Mylonakis E., Poulakou G., Young B.E., Vock D.M., Siegel L., Engen N., Grandits G., Mosaly N.R., Vekstein A.M. (2022). Efficacy and Safety of Ensovibep for Adults Hospitalized With COVID-19: A Randomized Controlled Trial. Ann. Intern. Med..

[22] Léger C., Di Meo T., Aumont-Nicaise M., Velours C., Durand D., Li de la Sierra-Gallay I., van Tilbeurgh H., Hildebrandt N., Desmadril M., Urvoas A. (2019). Ligand-induced conformational switch in an artificial bidomain protein scaffold. Sci. Rep..

[23] Peng Y., Zeng W., Ye H., Han K.H., Dharmarajan V., Novick S., Wilson I.A., Griffin P.R., Friedman J.M., Lerner R.A. (2015). A General Method for Insertion of Functional Proteins within Proteins via Combinatorial Selection of Permissive Junctions. Chem. Biol..

[24] Kim T.Y., Seo H.D., Lee J.J., Kang J.A., Kim W.S., Kim H.M., Song H.Y., Park J.M., Lee D.E., Kim H.S. (2018). A dimeric form of a small-sized protein binder exhibits enhanced anti-tumor activity through prolonged blood circulation. J. Control. Release.

[25] Gupta R.K., Gregson J., Parkin N., Haile-Selassie H., Tanuri A., Andrade Forero L., Kaleebu P., Watera C., Aghokeng A., Mutenda N. (2018). HIV-1 drug resistance before initiation or re-initiation of first-line antiretroviral therapy in low-income and middle-income countries: A systematic review and meta-regression analysis. Lancet Infect. Dis..

[26] Praditwongwan W., Chuankhayan P., Saoin S., Wisitponchai T., Lee V.S., Nangola S., Hong S.S., Minard P., Boulanger P., Chen C.J. (2014). Crystal structure of an antiviral ankyrin targeting the HIV-1 capsid and molecular modeling of the ankyrin-capsid complex. J. Comput.-Aided Mol. Des..

[27] Waterhouse A., Bertoni M., Bienert S., Studer G., Tauriello G., Gumienny R., Heer F.T., de Beer T.A.P., Rempfer C., Bordoli L. (2018). SWISS-MODEL: Homology modelling of protein structures and complexes. Nucleic Acids Res..

[28] Wilkinson I.C., Hall C.J., Veverka V., Shi J.Y., Muskett F.W., Stephens P.E., Taylor R.J., Henry A.J., Carr M.D. (2009). High Resolution NMR-based Model for the Structure of a scFv-IL-1β Complex. J. Biol. Chem..

[29] MacKerell A.D., Bashford D., Bellott M., Dunbrack R.L., Evanseck J.D., Field M.J., Fischer S., Gao J., Guo H., Ha S. (1998). All-atom empirical potential for molecular modeling and dynamics studies of proteins. J. Phys. Chem. B.

[30] Phillips J.C., Hardy D.J., Maia J.D.C., Stone J.E., Ribeiro J.V., Bernardi R.C., Buch R., Fiorin G., Hénin J., Jiang W. (2020). Scalable molecular dynamics on CPU and GPU architectures with NAMD. J. Chem. Phys..

