# Supplementary material for: Dimeric Ankyrin with Inverted Module Promotes Bifunctional Property in Capturing Capsid to Impede HIV-1 Replication
Source: Int J Mol Sci. 2023 Mar 9;24(6):5266. doi: 10.3390/ijms24065266 (PMC10048781; doi:10.3390/ijms24065266)
Supplement: Supplementary file 1 [file ijms-24-05266-s001.zip › ijms-2236196-supplementary.pdf]

Supplementary Material

Supplementary Figure

A

Ank^{GAG}1D4$_{NC-NC}$

6XHis-tag · N-cap

MRGSHHHHHHGSAAADLGKKLLEAARAGQDDEVRLLLEHGADVNAR

1st repeat

DSIGSTPLHLAAYYGHLEIVRLLLEHGADVNAR

2nd repeat

DSTGTTPLHYAARLGHLEIVRLLLEHGADVNAR

3rd repeat

DAMGWTPLHLAAKKGHLEIVRLLLKHGADVNAN

C-cap · Flexible Linker

DHFGKTAFDISIDNGNEDLAEILLQGGGGSGGGGSGGGGSGGGGSTS

N-cap

DLGKKLLEAARAGQDDEVRLLLEHGADVNAR

1st repeat

DSIGSTPLHLAAYYGHLEIVRLLLEHGADVNAR

2nd repeat

DSTGTTPLHYAARLGHLEIVRLLLEHGADVNAR

3rd repeat

DAMGWTPLHLAAKKGHLETVRLLLKHGADVDAN

C-cap

DHFGKTAFDISIDNGNEDLAEILQSLIS

B

Ank^{GAG}1D4$_{NC-CN}$

6XHis-tag · N-cap

MRGSHHHHHHGSAAADLGKKLLEAARAGQDDEVRLLLEHGADVNAR

1st repeat

DSIGSTPLHLAAYYGHLEIVRLLLEHGADVNAR

2nd repeat

DSTGTTPLHYAARLGHLEIVRLLLEHGADVNAR

3rd repeat

DAMGWTPLHLAAKKGHLEIVRLLLKHGADVNAN

C-cap · Flexible Linker

DHFGKTAFDISIDNGNEDLAEILLQGGGGSGGGGSGGGGSGGGGSTS

C-cap

LIEALDENGNDISIDFATKGFHD

1st repeat

NANVDAGHKLLLRVIELHGKKAALHLPTWGMAD

2nd repeat

RANVDAGHELLLRVIELHGLRAAYHLPTTGTSD

3rd repeat

RANVDAGHELLLRVIELHGYYAALHLPTSGISD

N-cap

RANVDAGHELLLRVEDDQGARAAELLKKGLD

Figure S1. Amino acid sequences of dimeric Ank^{GAG}1D4. The design of dimer platforms featured the different orientations of monomeric Ank^{GAG}1D4, consisting of three repetitive domains (blue) connected by a flexible linker (green) and flanked by N- and C-terminal capping repeats (red). (A) Ank^{GAG}1D4$_{NC-NC}$, the C-terminus of the Ank^{GAG}1D4 module is connected to the N-terminus of the

second module. (B) $Ank^{GAG}1D4_{NC\text{-}CN}$, the C-terminus of the $Ank^{GAG}1D4$ module is connected to the C-terminus of the second module.
